# HSPA12B: a novel facilitator of lung tumor growth

**DOI:** 10.18632/oncotarget.3533

**Published:** 2015-03-12

**Authors:** He Ma, Ting Lu, Xiaojin Zhang, Chuanfu Li, Jingwei Xiong, Lei Huang, Ping Liu, Yuehua Li, Li Liu, Zhengnian Ding

**Affiliations:** ^1^ Department of Anesthesiology, First Affiliated Hospital with Nanjing Medical University, Nanjing, China; ^2^ Department of Geriatrics, First Affiliated Hospital with Nanjing Medical University, Nanjing, China; ^3^ Department of Surgery, East Tennessee State University, Johnson City, TN, USA; ^4^ Department of Oncology, First Affiliated Hospital with Nanjing Medical University, Nanjing, China; ^5^ Department of Pathophysiology, Nanjing Medical University, Nanjing, China

**Keywords:** heat shock protein A12B, lung cancer, angiogenesis, proliferation, apoptosis

## Abstract

Lung tumor progression is regulated by proangiogenic factors. Heat shock protein A12B (HSPA12B) is a recently identified regulator of expression of proangiogenic factors. However, whether HSPA12B plays a role in lung tumor growth is unknown. To address this question, transgenic mice overexpressing HSPA12B (Tg) and wild-type littermates (WT) were implanted with Lewis lung cancer cells to induce lung tumorigenesis. Tg mice showed significantly higher number and bigger size of tumors than WT mice. Tg tumors exhibited increased angiogenesis and proliferation while reduced apoptosis compared with WT tumors. Interestingly, a significantly enhanced upregulation of Cox-2 was detected in Tg tumors than in WT tumors. Also, Tg tumors demonstrated upregulation of VEGF and angiopoietin-1, downregulation of AKAP12, and increased eNOS phosphorylation compared with WT tumors. Celecoxib, a selective Cox-2 inhibitor, suppressed the HSPA12B-induced increase in lung tumor burden. Moreover, celecoxib decreased angiogenesis and proliferation whereas increased apoptosis in Tg tumors. Additionally, celecoxib reduced angiopoietin-1 expression and eNOS phosphorylation but increased AKAP12 levels in Tg tumors. Our results indicate that HSPA12B stimulates lung tumor growth via a Cox-2-dependent mechanism. The present study identified HSPA12B as a novel facilitator of lung tumor growth and a potential therapeutic target for the treatment of lung cancer.

## INTRODUCTION

Lung cancer is the leading cause of cancer-related death in humans worldwide, with an overall 5 year survival rate of approximately 15% [1, 2]. Despite advances in the diagnosis and treatment of lung cancer, only modest improvements in patient survival have been achieved in the past 25 years [1, 2]. A better understanding of the mechanisms underlying lung cancer development and progression is urgently needed for the design of novel therapeutic modalities such as molecular targeted therapies for the treatment of this disease.

Heat shock protein A12B (HSPA12B) is a distant member of the mammalian heat shock protein 70 (Hsp70) family because HSPA12B contains an atypical Hsp70 ATPase domain [3]. HSPA12B is expressed specifically in endothelial cells, which differs from the ubiquitous expression of other Hsp70 family members such as HSPA1A, HSPA1B, HSPA5 and HSPA8 [4-7]. This unique distribution pattern suggests the possible involvement of HSPA12B in the pathogenesis of endothelium-associated events. Indeed, previous studies from our group show that overexpression of HSPA12B protects the heart and brain from ischemic injury [5, 8]. Moreover, HSPA12B attenuates cardiac injury during endotoxemia [7]. These effects of HSPA12B are mediated by the regulation of the expression of proangiogenic factors such as Cox-2, VEGF, angiopoietin-1 (Ang-1) and eNOS [5-9], which play important roles in tumor progression [1, 2, 10, 11]. However, whether HSPA12B participates in lung cancer growth is unknown.

To address this question, a lung cancer model was generated by implanting Lewis lung cancer cells (LLCs) into transgenic mice overexpressing the HSPA12B gene (Tg) and wild-type control mice (WT). Overexpression of HSPA12B stimulated lung tumor growth, and this effect was mediated by Cox-2-dependent increases of angiogenesis and cell proliferation and decrease of cell apoptosis in lung tumors. The present study identified HSPA12B as a novel facilitator of lung tumor growth, which suggests that targeting HSPA12B could be a therapeutic strategy for the treatment of lung cancer.

## RESULTS

### HSPA12B is expressed in pulmonary endothelial cells

**Figure 1:**
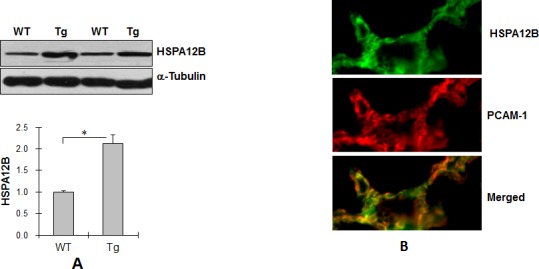

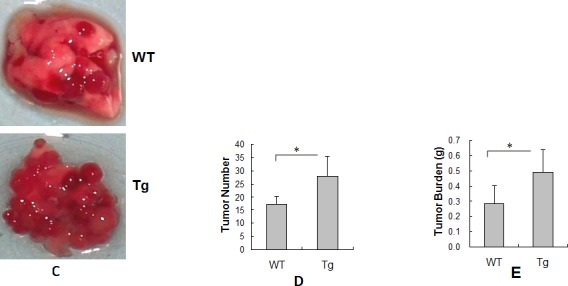
Endothelial HSPA12B facilitated lung tumor growth (A) Immunoblotting for HSPA12B. Lung tissues from WT and Tg mice (8-week old) were prepared for immunoblotting for HSPA12B. The same membrane was blotted with α-Tubulin to serve as a loading control. *P<0.01, n=6 per group. (B) Immunofluorescence for HSPA12B. Lung tissues from Tg mice (8-week old) were prepared for cryosectioning. The immunofluorescence staining for HSPA12B and PCAM-1 was performed. Note that HSPA12B (FITC) was colocalized with PCAM-1 (Cy3). Representative images from three independent experiments are shown. (C) Tumor formation. WT and Tg mice were implanted with LLCs to induce lung tumorigenesis. Lung tumor formation was examined and photographed 18 days after LLCs implantation. Note that Tg mice had a higher number and bigger size of tumors than WT mice. n=7- 9 per group. (D) Tumor number. WT and Tg mice were implanted with LLCs to induce lung tumorigenesis. Eighteen days later, the tumors were isolated from lungs for tumor number counting. *P<0.01, n=7- 9 per group. (E) Tumor Burden. WT and Tg mice were implanted with LLCs to induce lung tumorigenesis. Eighteen days later, the tumors were isolated from lungs for tumor burden weighing. *P<0.01, n=7- 9 per group. All quantitative data are expressed as means ± SD.

To investigate the roles of HSPA12B in lung cancer progression, we generated HSPA12B Tg mice. As shown in Figure 1A, HSPA12B protein levels were significantly higher (212.6%) in Tg lung tissues than in WT controls (*P <* 0.01). Immunofluorescence staining showed that HSPA12B (green) in Tg lung sections colocalized with PCAM-1 (red), a marker of endothelial cells (Figure 1B). Collectively, the results suggest that HSPA12B was overexpressed in pulmonary endothelial cells in Tg mice.

### HSPA12B facilitates lung tumor growth

Lung tumorigenesis was induced by implantation with LLCs by caudal vein injection in Tg and WT mice. Tumor formation was analyzed 18 days after LLCs implantation. As shown in Figure 1C, Tg mice had a higher number and bigger size of tumors than WT mice. The average tumor number was 28 in Tg and 17 in WT mice (Figure 1D). The tumor burden was 0.493 g in Tg and 0.285 g in WT mice (Figure 1E). Therefore, tumor number and tumor burden were 64.9% and 73.3% higher, respectively, in Tg than in WT mice (*P <* 0.01).

### HSPA12B increases angiogenesis in lung tumors

**Figure 2:**
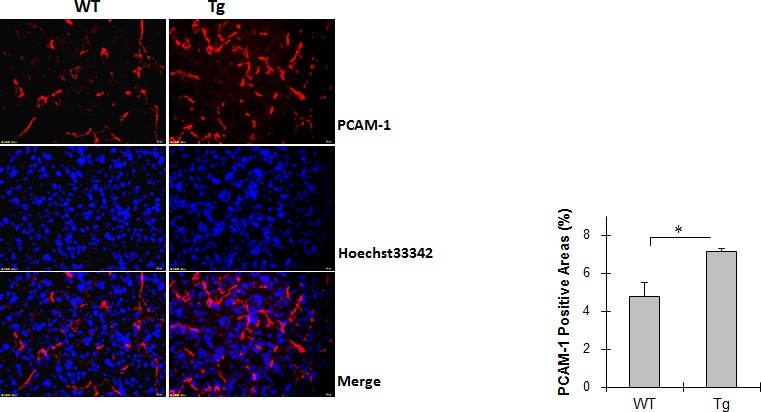
HSPA12B increased angiogenesis in lung tumors Lung tumor tissues were collected 18 days after LLCs implantation. Cryosections were prepared for immunostaining for PCAM-1. Hoechst 33342 was used to counter staining nuclei. The fluorescence images in tumors was observed and captured using a fluorescent microscope at a magnification of 400 ×. The quantitative data are expressed as means ± SD. *P<0.01, n=4-5 per group.

Angiogenesis in lung tumors was evaluated by immunostaining for PCAM-1. As shown in Figure 2, the percentage of PCAM-1-positive areas was 4.8% in WT tumor and 7.1% in Tg tumors. Thus, 49.3% more PCAM-1 positive areas were presented in Tg tumors than that in WT tumors (*P <* 0.01).

### HSPA12B inhibits apoptosis in lung tumors

**Figure 3:**
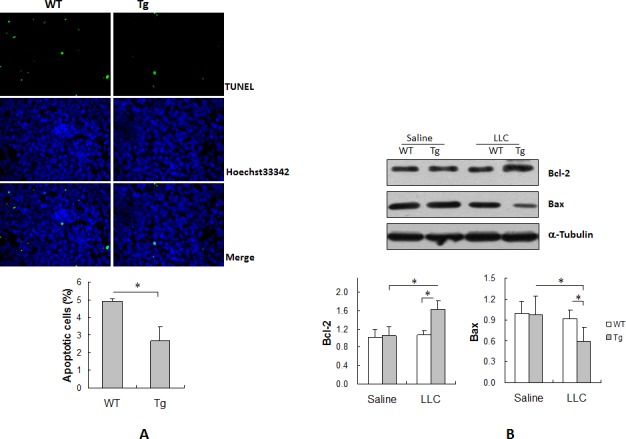
HSPA12B suppressed apoptosis in lung tumors Lung tumor tissues were collected 18 days after LLCs implantation for the following experiments. (A) TUNEL assay. Paraffin-embedded sections were prepared. A TUNEL assay was performed to detect apoptosis. Hoechst 33342 reagent was used to counterstain the nuclei. TUNEL-positive cells in tumors were observed using a fluorescent microscope at a magnification of 400 ×. *P<0.01, n=3-4 per group. (B) Immunoblotting analysis for Bcl-2 and Bax. Protein extracts were prepared for immunoblotting for Bcl-2 and Bax. The same membrane was blotted with α-Tubulin to serve as a loading control. *P<0.01, n=4-6 per group. All quantitative data are expressed as means ± SD.

Apoptosis of tumor cells is an important determinant of tumor load [12]. We therefore examined apoptosis in lung tumors by TUNEL staining. As shown in Figure 3A, the rate of apoptosis was 4.9% in WT tumors and 2.7% in Tg tumors. Interestingly, the rate of apoptosis was 45.8% lower in Tg than in WT tumors (*P <* 0.01).

Figure 3B shows the levels of expression of the antiapoptotic protein Bcl-2 and the proapoptotic protein Bax in lung tumors. The lung tissues from saline-treated mice served as normal controls. Bcl-2 and Bax levels were comparable between WT tumors and WT controls. However, a significant increase in Bcl-2 levels and decrease in Bax levels was detected in Tg tumors compared with Tg controls (*P <* 0.01). Importantly, Bcl-2 levels were significantly higher by 52.2% and Bax levels were significantly lower by 36.2% in Tg tumors than in WT tumors (*P <* 0.01). No significant difference in Bcl-2 or Bax levels was observed between the two normal controls.

### HSPA12B increases tumor cell proliferation

**Figure 4:**
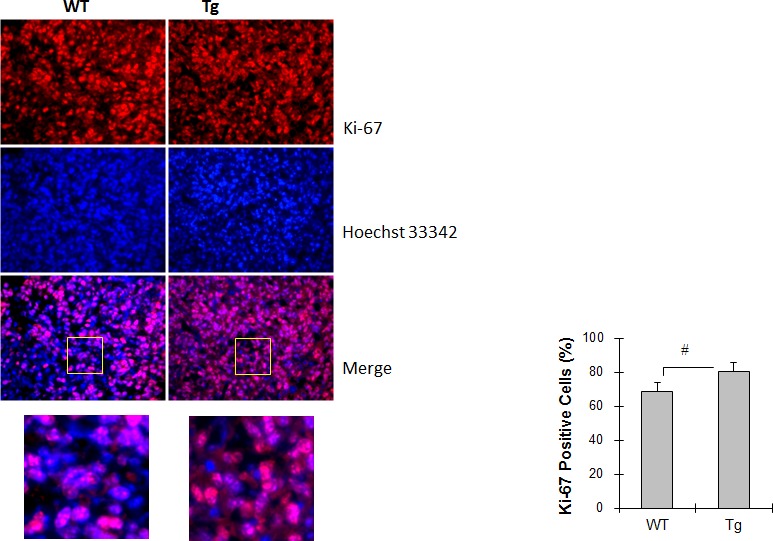
HSPA12B increased cell proliferation in lung tumors Lung tumor tissues were collected 18 days after LLCs implantation for paraffin-embedded sectioning. Slides were subjected to immunofluorescence staining for Ki-67 (Cy3, red). Hoechst 33342 reagent was used to counterstain the nuclei. Images were observed using a fluorescent microscope at a magnification of 400×. Higher magnification images of the boxed areas are shown in the down panels. The quantitative data are expressed as means ± SD. #*P<*0.05, n=4 per group.

To determine the role of tumor cell proliferation in the HSPA12B stimulation of lung tumor growth, cells were stained with Ki-67 as an indicator of cell proliferation and analyzed by immunofluorescence. The percentage of Ki-67-positive cells was 16.8% higher in Tg tumors than in WT tumors (80.2 ± 5.4% vs. 68.7 ± 5.0%, *P <* 0.05) (Figure 4).

### HSPA12B upregulates the expression of VEGF and Ang-1 and increases the phosphorylation of eNOS in lung tumors

**Figure 5:**
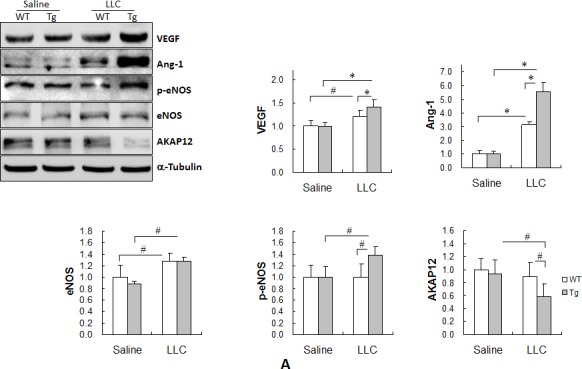

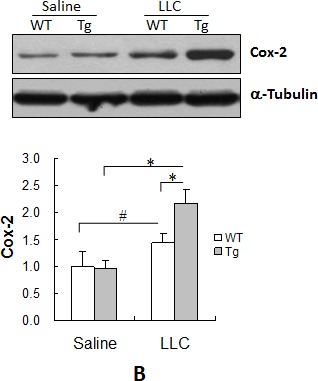
HSPA12B upregulated the expression of Cox-2, VEGF and Ang-1, increased the phosphorylation of eNOS, while decreased expression of AKAP12 in lung tumors Lung tumor tissues were collected 18 days after LLCs implantation. Protein extracts were prepared for immunoblotting with the indicated antibodies. The same membrane was blotted with α-Tubulin to serve as a loading control. All quantitative data are expressed as means ± SD. *P<0.01 and #*P<*0.05, n=4-6 per group.

We recently reported that HSPA12B upregulates the expression of proangiogenic factors (e.g., VEGF, Ang-1 and eNOS) in the ischemic myocardium [5]. Because these proangiogenic factors play important roles in the regulation of angiogenesis, apoptosis proliferation [5, 10, 13, 14], their expression levels were examined in lung tumors. As shown in Figure 5A, the levels of VEGF, Ang-1 and eNOS were significantly increased in both WT and Tg tumors compared with the genotype-matched normal controls (*P <* 0.01 or *P <* 0.05), with significantly higher levels of VEGF and Ang-1 (16.7% and 78.2%, respectively) in Tg than in WT tumors (*P <* 0.01). Although eNOS levels were comparable between WT and Tg tumors, phospho-eNOS (p-eNOS) levels were 37.0% higher in Tg tumors than in WT tumors (*P <* 0.05).

### HSPA12B decreases AKAP12 levels in lung tumors

The levels of A kinase anchor protein 12 (AKAP12), which exerts tumor suppressor activity [15, 16], were significantly lower in Tg tumors than in normal Tg lung tissues (*P <* 0.05) (Figure 4A). Moreover, Tg tumors exhibited significantly lower levels of AKAP12 by 38.5% compared with WT tumors (*P <* 0.05). AKAP12 levels were slightly lower in WT tumors than in normal WT lung tissues, although the difference did not reach statistical significance.

### HSPA12B enhances Cox-2 upregulation in lung tumors

Cox-2 plays critical roles in lung tumor progression [12, 17]. As shown in Figure 5B, Cox-2 levels were increased by 43.3% in WT tumors and 129.1% in Tg tumors compared with the genotype-matched normal lungs (*P <* 0.01 or *P <* 0.05), and they were 51.6% higher in Tg tumors than in WT tumors (*P <* 0.01) (Figure 5B). No significant difference in the Cox-2 level was observed between normal WT and Tg lungs.

### Inhibition of Cox-2 with celecoxib abolishes the HSPA12B-induced facilitation of lung tumor growth

**Figure 6:**
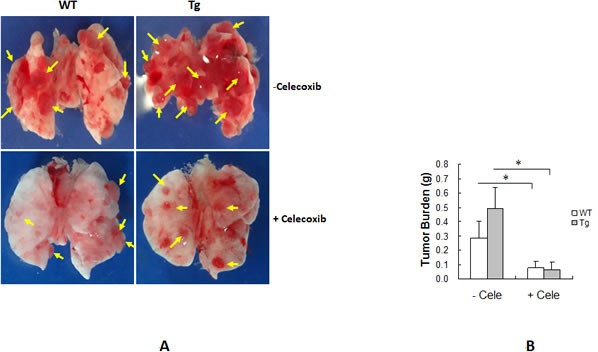
Cox-2 inhibitor celecoxib suppressed HSPA12B-induced stimulation of lung tumor growth WT and Tg mice were administrated with celecoxib (cele), a selective inhibitor for Cox-2, after LLCs implantation. Lungs were isolated and photographed 18 days after LLCs implantation. Representative images from six independent experiments are shown (A). Tumors were subsequently isolated from lungs for tumor burden weighing (B). The quantitative data are expressed as means ± SD. *P<0.01, n=6 per group.

To further examine the role of Cox-2 in the HSPA12B-induced facilitation of lung tumor growth, mice were treated with the selective Cox-2 inhibitor celecoxib after LLCs implantation. As shown in Figure 6A, tumor size was reduced in WT and Tg mice treated with celecoxib compared to the corresponding tumors in untreated mice. Tumor burden was significantly decreased by 72.0% in WT and 87.0% in Tg mice following celecoxib administration compared with the corresponding untreated tumors (*P <* 0.01) (Figure 6B). Importantly, no significant difference in tumor burden was observed between WT and Tg tumors following celecoxib treatment (0.080 ± 0.041 g vs. 0.064 ± 0.054 g, *P >* 0.05). These results suggest that celecoxib abolished the HSPA12B-induced facilitation of lung tumor growth.

### Celecoxib decreases angiogenesis in Tg tumors

**Figure 7:**
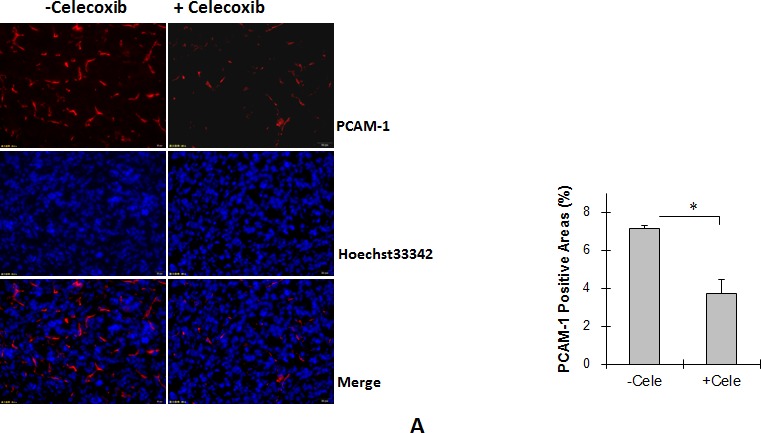

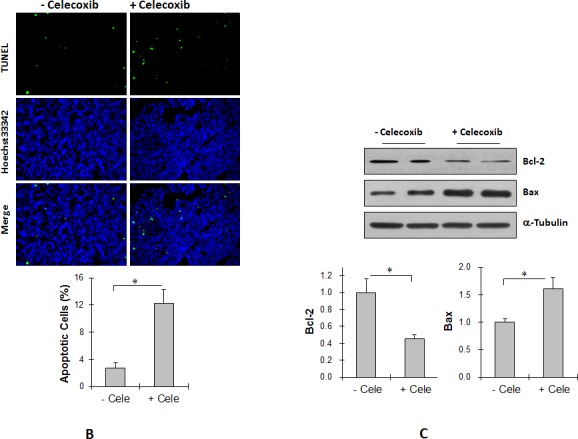

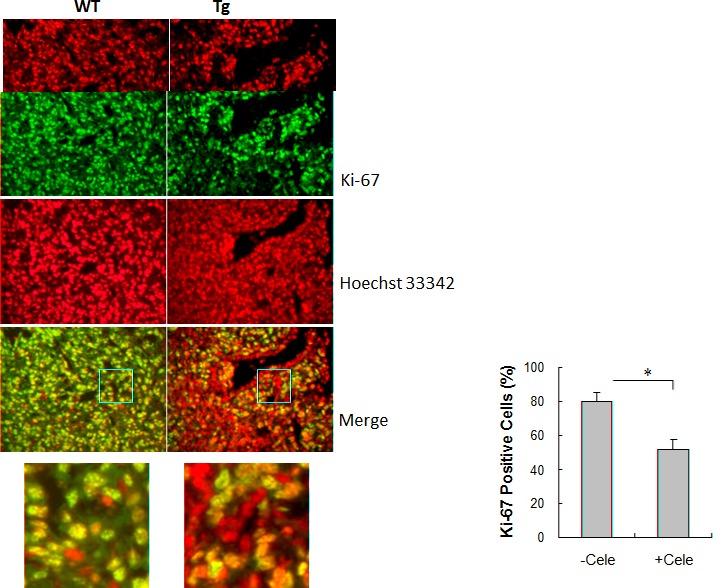
Cox-2 inhibitor celecoxib reduced angiogenesis and proliferation, and increased apoptosis in Tg lung tumors (A) Tg mice were administrated with celecoxib (cele) after LLCs implantation. Lung tumor tissues were collected 18 days after LLCs implantation for the following experiments. Angiogenesis. Cryosections were prepared for immunostaining against PCAM-1. Hoechst 33342 reagent was used to counterstain the nuclei. PCAM-1-positive areas in tumors were observed and calculated using a fluorescent microscope at a magnification of 400 ×. *P<0.01, n=4 per group. (B) TUNEL assay. Paraffin-embedded sections were prepared. A TUNEL assay was performed to detect apoptosis. Hoechst 33342 reagent was used to counterstain the nuclei. TUNEL-positive cells in tumors were observed using a fluorescent microscope at a magnification of 400 ×. *P<0.01, n=3-4 per group. (C) Immunoblotting for Bcl-2 and Bax. Protein extracts were prepared for immunoblotting for Bcl-2 and Bax. The same membrane was blotted with α-Tubulin to serve as a loading control. *P<0.01, n=4-8 per group. (D) Immunofluorescence staining for Ki-67. Paraffin-embedded sections were subjected to immunofluorescence staining for Ki-67 (Cy3, red). Hoechst 33342 reagent was used to counterstain the nuclei. Images were observed using a fluorescent microscope at a magnification of 400×. Higher magnification images of the boxed areas are shown in the down panels. *P<0.01, n=4 per group. All quantitative data are expressed as means ± SD.

As shown in Figure 7A, celecoxib treatment significantly decreased PCAM-1-positive areas by 48.2% in Tg tumors compared with untreated Tg tumors (3.48 ± 0.76% vs. 7.11 ± 0.15%, *P <* 0.01).

### Celecoxib increases cell apoptosis in Tg tumors

As shown in Figure 7B, a 360.5% increase in the rate of apoptosis was observed in Tg tumors treated with celecoxib compared with untreated Tg tumors (*P <* 0.01). The levels of Bcl-2 decreased significantly by 52.0% whereas Bax levels increased by 34.4% in Tg tumors treated with celecoxib compared with untreated Tg tumors (*P <* 0.01) (Figure 7C).

### Celecoxib suppresses cell proliferation in Tg tumors

Figure 7D shows the rate of Ki-67-positive cells in Tg lung tumors treated with or without celecoxib. Celecoxib treatment significantly decreased the percentage of Ki-67-positive cells in Tg tumors by 34.9% compared with that in untreated Tg tumors (*P <* 0.01).

### Celecoxib reduces Ang-1 expression and eNOS phosphorylation in Tg tumors

**Figure 8:**
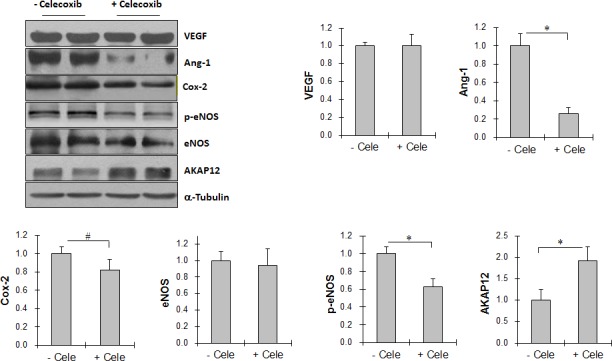
Cox-2 inhibitor celecoxib decreased Ang-1 expression, reduced eNOS activation, and increased AKAP12 expression in Tg lung tumors Tg mice were administrated with celecoxib (cele) after LLCs implantation. Tumor tissues were collected 18 days after LLCs implantation for immunoblotting analysis with the indicated antibodeis. The same membrane was blotted with α-Tubulin to serve as a loading control. All quantitative data are expressed as means ± SD. *P<0.01, n=4-8 per group.

As shown in Figure 8, Ang-1 levels were significantly reduced by 73.9% in Tg tumors treated with celecoxib compared with untreated Tg tumors (*P <* 0.01), whereas celecoxib had no significant effect on VEGF and eNOS expression. The levels of phosphorylated eNOS in Tg tumors were significantly reduced by 37.6% by celecoxib treatment compared with that in untreated Tg tumors (*P <* 0.01). Celecoxib administration significantly reduced Cox-2 levels by 18.4% in Tg tumors (*P <* 0.05).

### Celecoxib increases AKAP12 expression in Tg tumors

In contrast to the effects of celecoxib on Ang-1 expression, AKAP12 expression levels were increased by 91.2% in Tg tumors treated with celecoxib compared with untreated Tg tumors (*P <* 0.01) (Figure 8).

## DISCUSSION

In the present study, we showed that HSPA12B in pulmonary endothelial cells stimulated lung cancer growth in mice by enhancing angiogenesis, increasing cell proliferation, and suppressing apoptosis in tumors via a Cox-2-dependent mechanism.

Recent evidence demonstrates that tumor growth is regulated by the cross-talk between endothelial cells and tumor cells [18, 19]. As an example, lung carcinoma cells when co-cultured with endothelial cells modify their cellular molecular features that encourage tumor cell survival through an undefined mechanism [18]. Knockdown of endothelial Dll4 stimulates the xenograft tumor growth of lung cancer cells through promoting the proliferation of neighboring cancer cells, and vice versa [19]. Acute depletion of endothelial β3-integrin transiently inhibits tumor growth and angiogenesis in mice [20]. Moreover, inhibition of endothelial FAK activity prevents tumor metastasis by enhancing barrier function [21]. These observations suggest an important role of endothelial events in the regulation of lung tumor progression. In this study, we provided first evidence for that HSPA12B, which expresses predominantly in endothelial cells, stimulated lung tumor growth as reflected by a significant increase in tumor number and tumor burden.

Solid tumor growth and progression is dependent on tumor-associated angiogenesis [22, 23]. Therefore, angiogenesis is a promising target for lung cancer. The only antiangiogenic agent currently approved for the treatment of lung cancer is the VEGF monoclonal antibody bevacizumab. However, bevacizumab only modestly improved survival time (2 months) in patients with advanced non-small cell lung cancer. Moreover, bevacizumab use was associated with increased treatment-related death [22, 24, 25]. Thus, further development of novel antiangiogenic strategies for lung cancer is strongly needed. In this study, we observed that HSPA12B increased angiogenesis in lung tumors, which showed positive correlations with lung tumor growth. In supporting this observation, previous studies by ours and others have demonstrated that HSPA12B increased tube formation in endothelial cells and angiogenesis in myocardium post-infarction [5, 9, 25]. Collectively, HSPA12B is a facilitator of lung cancer-associated angiogenesis. Further studies are needed to investigate whether targeted suppressing HSPA12B would limit the growth and progression of lung cancer.

HSPA12B Tg lung tumors showed a significantly decreased rate of apoptosis, which was negatively correlated with lung tumor growth. This was supported by an increase in Bcl-2 levels and a decrease in Bax levels in Tg tumors compared with those in WT tumors. Apoptosis is an important mechanism limiting lung tumor progression [26-28]. For example, failure to efficiently induce apoptosis contributes to cisplatin resistance in lung cancer [27]. On the other hand, apoptosis sensitization mediates the tumor suppressor effects of rapamycin, decitabine and vorinostat [26, 28]. Taken together, our results indicate that the suppression of apoptosis contributed to the HSPA12B-induced growth of lung tumors.

The proliferation rate of cancer cells is positively correlated with tumor progression [26, 29, 30]. An increased proliferation rate is associated with the CD97-promoted progression of thyroid cancer, whereas the suppression of tumor cell proliferation mediates the inhibitory effects of PDE4i on tumor xenograft growth [31, 32]. In the present study, Tg lung tumors showed an increased proliferation rate compared to WT tumors, as indicated by the Ki-67 positive staining. Collectively, these results suggest that the increase in cell proliferation contributed to the growth of Tg lung tumors.

Apoptosis and proliferation of cancer cells are regulated by proangiogenic factors such as Cox-2, VEGF, Ang-1 and eNOS [1, 11, 33-35]. Ho et al. showed that suppression of Cox-2 activity mediates the IL-27-induced reduction of proliferation and restriction of lung tumorigenicity [1]. Baek et al. reported that VEGF enhances tumor survivability via suppression of apoptosis [10]. Human lung carcinoma tissues have significantly higher levels of Ang-1 and its receptor Tie2 than adjacent noncancerous tissues [36]. Moreover, eNOS has been shown to protect prostate cancer cells from TRAIL-induced apoptosis [35]. In the present study, the levels of Cox-2, VEGF, Ang-1 and phosphorylated eNOS were significantly higher in Tg lung tumors than in WT lung tumors. Consistent with these results, we recently reported that overexpression of HSPA12B upregulates VEGF and Ang-1 in ischemic hearts, and HSPA12B upregulates Ang-1 expression in ischemia/reperfusion brains and endotoxemic hearts [5, 7, 8, 35]. Taken together, these findings suggest that proangiogenic factors are involved in the HSPA12B-induced decrease in apoptosis and increase in cell proliferation in lung tumors, which in turn promoted lung tumor growth.

Cox-2 plays critical roles in lung tumor progression [12, 17]. We observed that HSPA12B enhanced Cox-2 upregulation in lung tumors. To determine the role of upregulated Cox-2 in the HSPA12B-induced stimulation of lung tumor growth, mice were treated with the selective Cox-2 inhibitor celecoxib after LLCs implantation. Celecoxib significantly reduced tumor burden in both WT and Tg mice. Most importantly, tumor burden was comparable between celecoxib-treated WT and celecoxib-treated Tg mice, suggesting that Cox-2 inhibition completely suppressed HSPA12B-induced tumor growth. In addition, the HSPA12B-induced increase in cell proliferation and decrease in apoptosis in lung tumors was abolished by Cox-2 inhibition. Furthermore, Cox-2 inhibition decreased Ang-1 expression levels, suppressed eNOS phosphorylation levels, and increased AKAP12 expression levels in HSPA12B Tg tumors compared to those in untreated Tg tumors. Previous studies showed that Cox-2 inhibition decreases Ang-1 expression in U-87MG cells and eNOS activation in damaged kidneys, which is consistent with the present results [37, 38]. Taken together, our results suggest that the HSPA12B-induced stimulation of lung tumor growth was mediated by Cox-2.

So far there is no direct evidence showing how HSPA12B regulates the expression of Cox-2, VEGF, Ang-1 and eNOS. However, evidence has suggested that AKAP12 may play a role in this regulation based on the following reasons. Firstly, AKAP12 has been shown to interact directly with HSPA12B on a yeast 2-hybrid system [9]. Secondly, AKAP12 is a kinase-scaffolding protein possessing angiogenic inhibition characters [39]. Overexpression of AKAP12 disrupts endothelial tube formation in Matrigel substrates, and vice versa [39]. Thirdly, AKAP12 negatively regulates the expression of VEGF, Ang-1 and eNOS [40, 41], which could be mediated by a downregulation of HIf-1α. Finally, though no direct evidence showing how HSPA12B regulates Cox-2 expression, VEGF may be involved in. Shtivelband and his colleagues have reported that administration of VEGF stimulated Cox-2 expression in human endothelial cells [42]. Consistent with this observation, other studies also demonstrated that VEGF serves as a regulator of Cox-2 expression [43]. On the other hand, Cox-2 can affect VEGF Production [44]. It is possible that HSPA12B downregulated AKAP12, which in turn increased VEGF expression and thereby stimulated Cox-2 expression.

In summary, the present study showed for the first time that HSPA12B promotes lung tumor growth through a mechanism involving Cox-2. Our data indicates that HSPA12B could be an alternative therapeutic target for the suppression of lung cancer progression.

## MATERIALS AND METHODS

### Antibodies and chemicals

Primary antibody against α-Tubulin was purchased from Sigma-Aldrich (St Louis, MO). The primary antibody against HSPA12B was a generous gift from Dr. Zhihua Han (East Tennessee State University) [9]. The remaining primary antibodies and the companies that supplied them were as follows: Bcl-2, Bax, Cox-2 and p-eNOS (Cell Signaling Technology, Beverly, MA); PCAM-1 and Ki-67 (BD Pharmingen, San Jose, CA); eNOS (BD Biosciences, Bedford, MA); and VEGF, Ang-1 and AKAP12 (Abcam, Cambridge, UK). Celecoxib was obtained from Pfizer Pharmaceuticals LLC (Barceloneta, PR). The TUNEL assay kit was from Promega (Madison, WI). The supersignal west pico chemiluminescent substrate was obtained from Pierce (Rockford, IL).

### Animals

Investigation has been conducted in accordance with the ethical standards and according to the national and international guidelines and has been approved by Nanjing University review board. Transgenic mice overexpressing the human *hspa12b* gene (Tg) driven by its own promoter were developed as described in our previous studies [5, 7]. Male littermates of HSPA12B Tg mice and wild type (WT) mice at 8-10 week of age were used in the present study. Mice were bred and maintained at the Model Animal Research Center of Nanjing University and maintained in the Animal Laboratory Resource Facility at Nanjing University.

### Lung tumorigenesis induction

Lung tumorigenesis in mice was induced by implantation with LLCs. LLCs were obtained from ATCC and maintained in DMEM supplemented with 10% FCS. LLCs were collected from 85% confluence cultures and implanted into mice (1.5 × 10^5^ cells/mice) via tail intravenous injection. The saline-injected mice served as normal controls. Lung tumor formation was evaluated 18 days after LLCs implantation. Briefly, the mice were sacrificed by an overdose of anesthesia (pentobarbital sodium 150 mg/kg, intraperitoneal injection) and cervical dislocation. Lungs were isolated and photographed after thoracotomy. Subsequently, the tumors were isolated from the lungs, counted and weighed.

For Cox-2 inhibition experiments, WT and Tg mice were fed with chows containing celecoxib (1.5 g/kg chow) after LLCs transplantation until the experiments were completed according to previously described methods [45].

### Immunofluorescence staining

For examination of colocalization of HSPA12B and PCAM-1, immunofluorescence experiments were performed following previously described protocols [5, 7]. Briefly, lung tissues were collected from adult Tg mice and prepared for cryosectioning. After blocking with 3.5% normal goat serum for 30 min, the cryosections were incubated with the appropriate primary antibodies overnight, washed and incubated with FITC- and/or Cy3-labelled secondary antibodies for 60 min. The sections were observed under a fluorescent microscope (Zeiss Ltd., Oberkochen, Germany) at a magnification of 400×.

For evaluation of angiogenesis in lung tumors, immunofluorescence staining for PCAM-1 was performed on cryosections of lung tumor tissues which were collected 18 days after LLCs implantation. The staining protocol was as same as mentioned above. The PCAM-1-positive staining in tumors was observed using a fluorescent microscope at a magnification of 400×. The percentages of PCAM-1-positive areas in more than six randomly selected tumor fields of each sample were measured using a computerized software (*Olympus, Japan*).

For evaluation of cell proliferation in lung tumors, immunofluorescence staining for Ki-67 was performed following previously described protocols with a minor modification [46]. Briefly, lung tumor tissues were collected 18 days after LLCs implantation and prepared for paraffin-embedded sectioning. Slides were deparaffinized and microwaved to retrieve antigen. After blocking with 3.5% normal goat serum, the cryosections were incubated with primary antibody for Ki-67 overnight followed by incubation with Cy3-labelled secondary antibody. Hoechst 33342 reagent was used to counterstain the nuclei. The number of Ki-67-positive cells in tumors was counted in more than six randomly selected fields using a fluorescent microscope at a magnification of 400× (Zeiss Ltd. Oberkochen, German). The percentage of Ki-67-stained nuclei over total nuclei was calculated.

### Apoptosis in tumors

Lung tumor tissues were collected 18 days after LLCs implantation and processed for paraffin-embedded sectioning. Apoptosis in tumors was examined using a TUNEL assay kit as described in our previous studies [5, 8]. The Hoechst 33342 reagent was used to counterstain the nuclei. The number of TUNEL-positive cells in tumors was counted in more than five randomly selected fields using a fluorescent microscope at a magnification of 400× (Zeiss Ltd. Oberkochen, German). The percentage of apoptotic cells over total cells was calculated.

### Immunoblotting analysis

Lung tumor tissues were collected 18 days after LLCs implantation. Lung tissues from saline-treated mice served as normal controls. The protein extracts were prepared and equal amount of proteins were separated by 10% SDS-PAGE and transferred onto Immobilon-P membranes (Millipore). The membranes were probed with the appropriate primary antibodies, followed by incubation with peroxidase-conjugated secondary antibodies. The signals were detected by enhanced chemiluminescence. To control for lane loading, the same membranes were probed with anti-α-Tubulin. The signals were quantified by scanning densitometry and the results from each experimental group were expressed as relative integrated intensity compared with that of controls.

### Statistical analysis

Results are expressed as mean ± standard deviation (X ± SD). Groups were compared using Student two-tailed unpaired *t* test or one-way analysis of variance analysis (ANOVA) followed by Tukey post hoc test, as appropriate with SPSS 13.0 software (SPSS Inc., Chicago, IL). Statistical significance was set at *P<* 0.05.

## SUPPLEMENTARY MATERIALS, FIGURES


