# Supplementary material for: HSPA12B: a novel facilitator of lung tumor growth
Source: Oncotarget. 2015 Mar 12;6(12):9924–36. doi: 10.18632/oncotarget.3533 (PMC4496407; doi:10.18632/oncotarget.3533)
Supplement: Supplementary file 1 [file oncotarget-06-9924-s001.pdf]

HSPA12B: a novel facilitator of lung tumor growth

Supplemental Material

Methods

1. Isolation and growth of human umbilical vein endothelial cells (HUVECs)

HUVECs were isolated from umbilical vein cords of normal pregnancies according to our previously described methods [1]. The cells in passage 2 to 5 were used for experiments. This study has been approved by the ethical committee of the First Affiliated Hospital with Nanjing Medical University (2012-SR-153).

2. Overexpression of HSPA12B in HUVECs

To achieve HSPA12B overexpression, HUVECs were infected with adenovirus containing human *hspa12b* expression full length cDNA which fused with *3 flags*. HUVECs infected with empty adenovirus were served as controls. The overexpression of HSPA12B was confirmed by immunoblot analysis. The endogenous HSAPA12B is 75 kD and the transfected HSPA12B is 78 kD consistent of 75 kD HSPA12B and 3 kD flags.

3. Examination of proliferation of Lewis lung cancer cells (LLCs)

The HUVECs that grown on transwell insert were infected with *hspa12b*– adenovirus to overexpress HSPA12B. The empty virus infected HUVECs served as controls. Forty-eight hours after infection, HUVECs were co-cultured with LLCs in transwell. MTT assay was performed to evaluate the proliferation of LLCs by MTT assay 12 h after co-culture according to our previous described method [2].

Results

1.Cox-1 expression levels in Tumors

Cox-1 expression levels were examined in WT and Tg tumors by immunoblot analysis. As shown in **Figure S1**, no significant difference was observed between WT and Tg tumors.

2. Overexpression of HSPA12B in HUVECs

To achieve HSPA12B overexpression, HUVECs were infected with *hspa12b*–adenovirus. HUVECs infected with empty adenovirus were served as controls. The overexpression of HSPA12B was confirmed by immunoblot analysis, which demonstrated an abrupt increase of HSPA12B in *hspa12b*-adenovirus infected HUVECs (**Figure S2**). HSPA12B was hardly detected in HUVECs infected with empty virus because too little proteins (10 μg) were loaded for analysis. It is true that endogenous

HSPA12B has been proved expressing in HUVECs by previous studies of ours and others [1, 3].

3. Release of HSPA12B into extracellular space of HUVECs

We then determine whether HSPA12B could be released into extracellular space form HUVECs. Forty-eight hours after virus infection, culture medium was collected and centrifuged at 3000 rpm. Supernatant with a volume of 40 µl was loaded on 10% SDS-PAGE for immunoblotting analysis against HSPA12B. The results demonstrated an appearance of HSPA12B in the culture medium of empty-virus infected control HUVECs, suggesting that HSPA12B was released into extracellular space from endothelial cells. Interestingly, the culture medium from HSPA12B-overexpresssed HUVECs demonstrated a significant increase of HSPA12B by 15.5-fold, in comparison with the medium from control HUVECs ($P<0.01$) (**Figure S3**).

4. Endothelial HSPA12B stimulates proliferation of LLCs

To determine whether endothelial HSPA12B could stimulate proliferation of LLCs, The HUVECs that grown in transwell insert were infected with *hspa12b* – adenovirus to overexpress HSPA12B. The empty virus infected HUVECs served as controls. Forty-eight hours after infection, HUVECs were co-cultured with LLCs in transwell. MTT assay was performed to evaluate the proliferation of LLCs 12 h after co-culture (**Figure S4)**. The results demonstrated that compared to LLCs co-cultured with control HUVECs,

the proliferation of LLCs co-cultured with HSPA12B-overexpressed HUVECs was significantly increased by 38.68% ($P<0.01$).

5. Expression of angiogenic factors in HUVECs and LLCs in co-culture system

The effects of HSPA12B on the expression of angiogenic factors in HUVECs and LLCs in co-culture system were examined. The HUVECs that grown in transwell insert were infected with *hspa12b*–adenovirus to overexpress HSPA12B. The empty virus infected HUVECs served as controls. Forty-eight hours after infection, HUVECs were co-cultured with LLCs in transwell. Cells were collected at 48 h after co-culture. Protein extracts were prepared for immunoblot analysis. **Figure S5** shows the results in HUVECs. Overexpression of HSPA12B increased Cox-2 by 5.5-fold, VEGF by 6.9-fold and Ang-1 by 0.46-fold, respectively, compared with control HUVECs ($P<0.01$). Conversely, AKAP12 was decreased in HSPA12B-overexpressed HUVECs compared with control HUVECs ($P<0.05$). **Figure S6** shows the results in LLCs. Only VEGF was upregulated by 3.4-fold whereas Cox-2 and Ang-1 remained unchanged in LLCs that co-cultured with HSPA12B-overexpressed HUVECs, compared with LLCs that co-cultured with control HUVECs.

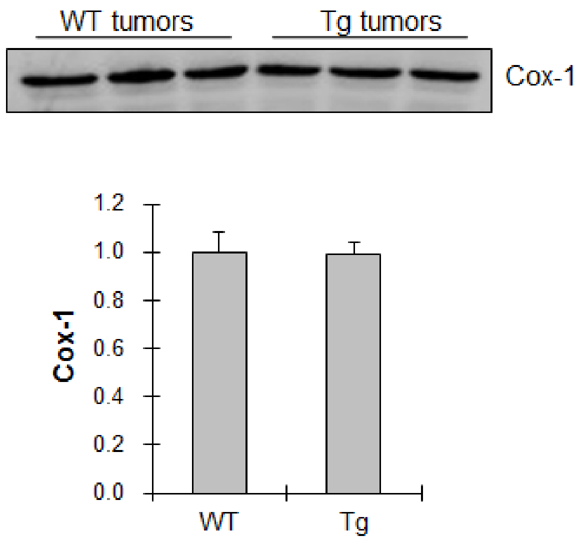

Figure S1: Cox-1 level examination.

Lung tumor tissues were collected 18 days after LLCs implantation in WT and Tg mice. Protein extracts were prepared for immunoblot analysis against Cox-1. All quantitative data are expressed as means ± SD. n=3 per group.

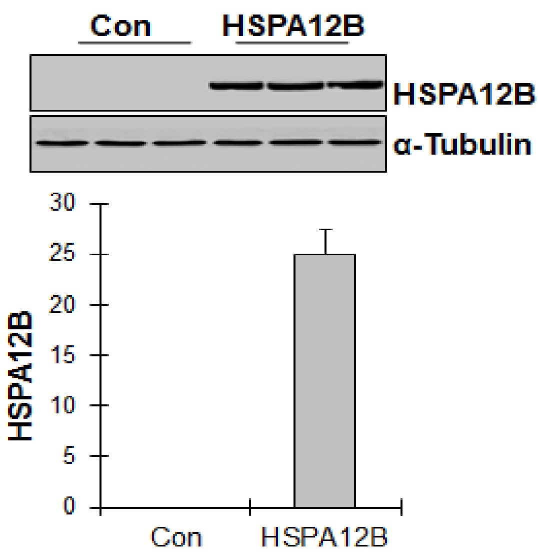

Figure S2: Overexpression of HSPA12B in HUVECs by *hspa12b*-adenovirus infection.

HUVECs were infected with adenovirus containing human *hspa12b* expression full length cDNA. HUVECs infected with empty adenovirus were served as controls. Cells were collected 48 h after infection for immunoblot analysis against HSPA12B. The same membrane was blotted with α-Tubulin to serve as a loading control. All quantitative data are expressed as means ± SD. n=4 per group.

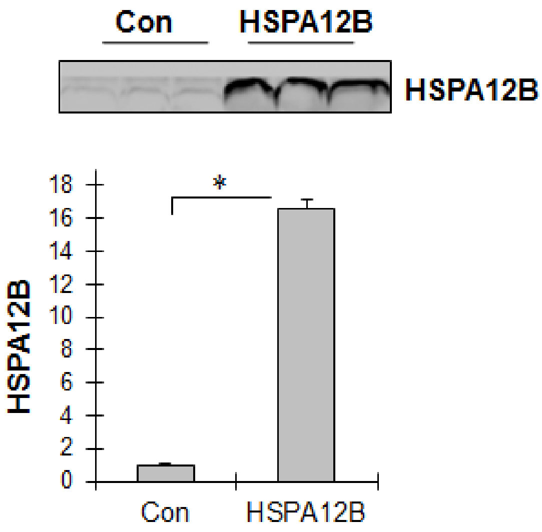

Figure S3: Examination of HSPA12B in extracellular space of HUVECs.

HUVECs were infected with *hspa12b*-adenovirus. HUVECs infected with empty adenovirus were served as controls. Culture medium was collected for immunoblot analysis against HSPA12B 48 h after infection. All quantitative data are expressed as means ± SD. **P*<0.01, n=3 per group.

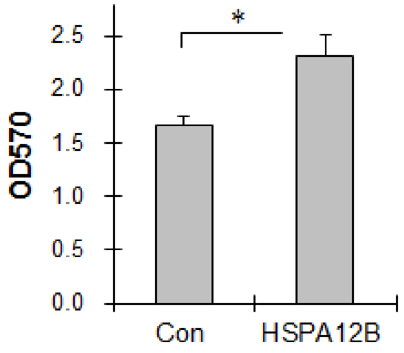

Figure S4: Effects of endothelial HSPA12B on the proliferation of LLCs

HUVECs that grown on transwell insert were infected with *hspa12b*-adenovirus. HUVECs infected with empty adenovirus were served as controls. Forty-eight hours after infection, HUVECs were co-cultured with LLCs in transwell. MTT assay was performed to evaluate the proliferation of LLCs after co-culture for 12 h. All quantitative data are expressed as means ± SD. *P<0.01, n=4 per group.

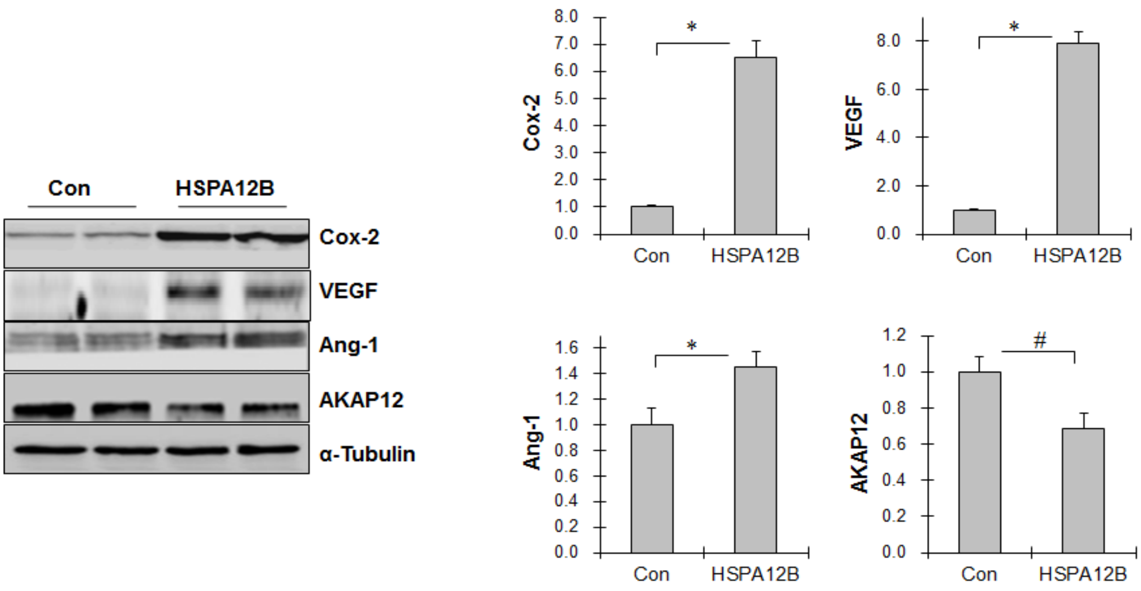

Figure S5: Expression of angiogenic factors in HUVECs in co-culture system

The HUVECs that grown in transwell insert were infected with *hspa12b* – adenovirus to overexpress HSPA12B. The empty virus infected HUVECs served as controls. Forty-eight hours after infection, HUVECs were co-cultured with LLCs in transwell. HUVECs were collected at 48 h after co-culture. Protein extracts were prepared for immunoblot analysis against the indicted primary antibodies. The same membrane was blotted with α-Tubulin to serve as a loading control. All quantitative data are expressed as means ± SD. $^{*}P<0.01$ and $^{\#}P<0.05$, n=3 per group.

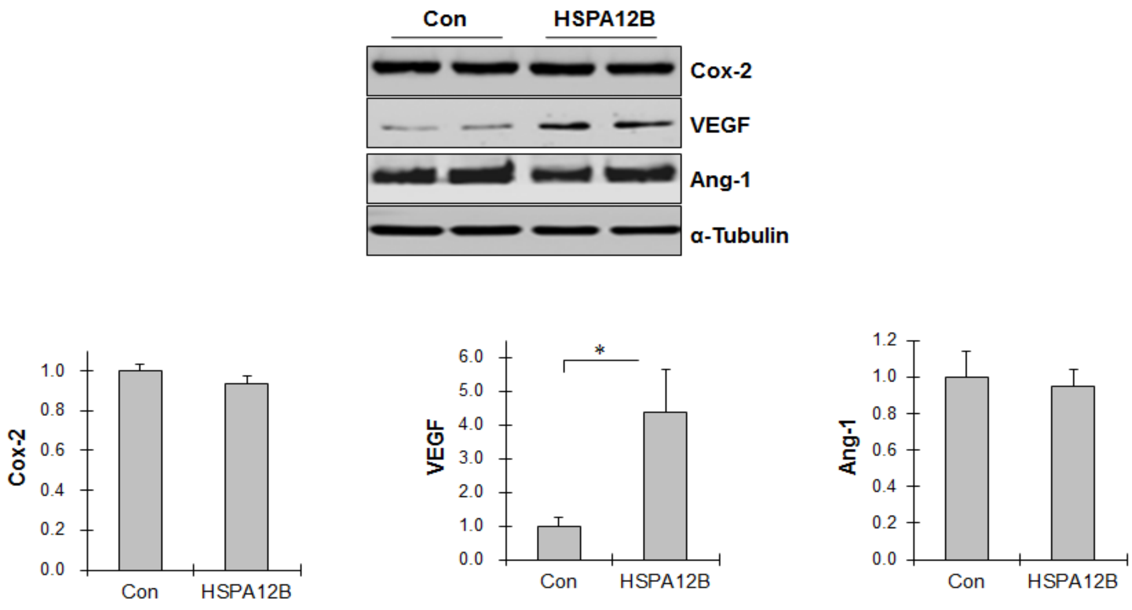

Figure S6: Expression of angiogenic factors in LLCs in co-culture system

The HUVECs that grown on transwell insert were infected with *hspa12b* –adenovirus to overexpress HSPA12B. The empty virus infected HUVECs served as controls. Forty-eight hours after infection, HUVECs were co-cultured with LLCs in transwell. LLCs were collected at 48 h after co-culture. Protein extracts were prepared for immunoblot analysis against the indicted primary antibodies. The same membrane was blotted with α-Tubulin to serve as a loading control. All quantitative data are expressed as means ± SD. *P<0.01, n=3 per group.

References

1. Li J, Zhang Y, Li C, Xie J, Liu Y, Zhu W, Zhang X, Jiang S, Liu L and Ding Z. HSPA12B attenuates cardiac dysfunction and remodelling after myocardial infarction through an eNOS-dependent mechanism. Cardiovascular research. 2013; 99(4):674-684.
2. Li R, Ma H, Zhang X, Li C, Xiong J, Lu T, Mao Y, Dai J, Liu L and Ding Z. Impaired Autophagosome Clearance Contributes to Local Anesthetic Bupivacaine-induced Myotoxicity in Mouse Myoblasts. Anesthesiology. 2015.
3. Hu G, Tang J, Zhang B, Lin Y, Hanai J, Galloway J, Bedell V, Bahary N, Han Z, Ramchandran R, Thisse B, Thisse C, Zon LI and Sukhatme VP. A novel endothelial-specific heat shock protein HspA12B is required in both zebrafish development and endothelial functions in vitro. Journal of cell science. 2006; 119(Pt 19):4117-4126.
